# Supplementary material for: The connectivity degree controls the difficulty in reservoir design of random boolean networks
Source: Front Comput Neurosci. 2024 Mar 14;18:1348138. doi: 10.3389/fncom.2024.1348138 (PMC10972873; doi:10.3389/fncom.2024.1348138)
Supplement: Supplementary file 1 [file Data_Sheet_1.pdf]

6 TRAINING

Both tasks follow the same protocol for training the readout weights:

- Reservoirs receive the input u for $D = 2000$ time steps.
- We discard the first 500 time steps.
- The training is then performed on the subsequent 1500 time steps. We concatenate the reservoir outputs in time, and use the optimization procedure defined in 2.1.

Each experiment consists in 40 values of $\sigma^\star$ per sign of b, and is performed for three values of $K = \{4, 8, 16\}$ and three values of $N = \{100, 1000, 10000\}$. For each value ($\sigma^\star$, K, N), 20 reservoirs are randomly generated, and each network is run 5 times with different randomly tossed inputs (i.e., $14, 400$ simulations). Each training is performed for 4000 epochs (with a total of $57, 600, 000$ training epochs).

6.1 The density is not a control parameter

Historically, the litterature on RBN studied the connectivity degree K, along with the number of neurons in the reservoir N. It has been shown that RBN reservoirs possess interesting computational properties for very sparse matrices, with at most $K = 25$ connections per neuron, according to (Büsing u. a., 2010). On the other hand, studies in ESN have explored the impact of another parameter, the density d (Hajnal und Lörincz, 2006; Krauss u. a., 2019a; Metzner und Krauss, 2022), which captures how many zeros there are in the adjacency matrix, ranging from zero for no connections, to one for fully connected reservoirs.

In the context of RBN reservoirs, the density is equal to $d = K/N$. It is worth noting that for the values of K and N used in this study, the corresponding values of d are ridiculously small, ranging from $0, 0004$ to 0.16. This stresses how much the RBN behaves differently since phase transitions occur at a fraction of what is observed in an ESN. It also suggests that K is a more natural choice for the control parameter of the RBN.

Nonetheless, this is not sufficient to rule out d as a good candidate for control parameters. Recalling that it is in the same fashion that μ and σ were shown to be dependent parameters and $\sigma^\star = \mu/\sigma$ was built in (Calvet u. a., 2023). If d is a control parameter, however, then all combinations of K and N leading to the same values of d should provide the exact same dynamics and performance in tasks, as is the case with $\sigma^\star$. To find if d is indeed a control parameter, we study the dynamics and performance of three couples of K and N giving the same value d:

$$d = K/N = 16/10000 = 8/5000 = 4/2500 = 0.0016$$

In Fig. 11, we show the attractor statistics in the three conditions, as a function of b. The first thing we notice is that all three tested conditions give very different attractor profiles. The width and the location of the critical regions are not the same, and the proportion of attractors also greatly varies, with lower K and N giving much more varied dynamics and wider critical regions. It is intriguing that in Fig. 4 and Fig. 8, diminishing K and N results in a somewhat comparable increase in the dynamics variability as well, but if K and N seem related, it is not by their ratio as proposed in the density parameter.

In addition, we show in Fig. 12 the performance in memory and prediction of 20 reservoirs selected with the same b_{opt} values, for each couple (N, K) (for more information on how these plots are obtained see Sec. 3.1.2). Following the dynamics analysis, even though each couple (N, K) possesses the same density, the performance varies.

When considering $b < 0$, smaller K consistently provides higher performance in all considered tasks. This is interesting because it is in spite of the fact that reservoirs are smaller. On the other hand, for $b > 0$, we have a different behaviour, first in the memory task, performances are close even though ($K = 4$, $N = 2500$) performs slightly better in the most difficult ($\tau = -14$) set-up. Second, in the prediction task, ($K = 4$, $N = 2500$) and ($K = 8$, $N = 5000$) give very similar performance, while the latter have reservoirs of twice the size. Surprisingly, even though ($K = 16$, $N = 10000$) has the biggest network, its performance is way below the two others.

As a consequence, for the same given density value obtained with various combinations of K and N, we obtain distinct dynamics statistics and performance. We conclude that the density is not a control parameter for RBN reservoirs, and one must study the effect of K and N separately, as two independent control parameters.

Regarding the objective of the paper, these results suggest that K has a stronger influence on the performance than N, as increasing the size of the network does not necessarily guarantee higher performance, but reducing K consistently improves them.

7 OPTIMAL BALANCES FOR PERFORMANCE

K	1	2	3	4	5	8	16
$b > 0$	0.024	0.024	0.052	0.029	0.077	0.149	0.179
$b < 0$	-0.099	-0.069	-0.057	-0.029	-0.049	-0.52	-0.99

Table 1. Table of the balance b_{opt} giving the best average performance at white-noise memory for $\delta = -18$ ($N = 10000$). The green highlight represents the optimal K value. Lower values of K typically give the lowest b_{opt} values, while increasing K tends to increase b_{opt} in absolute value.

K	1	2	3	4	5	8	16
$b > 0$	0.95	0.19	0.068	0.024	0.098	0.15	0.19
$b < 0$	-0.24	-0.20	-0.12	-0.047	-0.42	-0.82	-0.95

Table 2. Table of the balance b_{opt} giving the best average performance at Mackey-Glass prediction for $\tau = 28$ ($N = 10000$). The green highlight represents the optimal K value, which typically gives the lowest b_{opt} values, while increasing K tends to increase b_{opt} in absolute value.

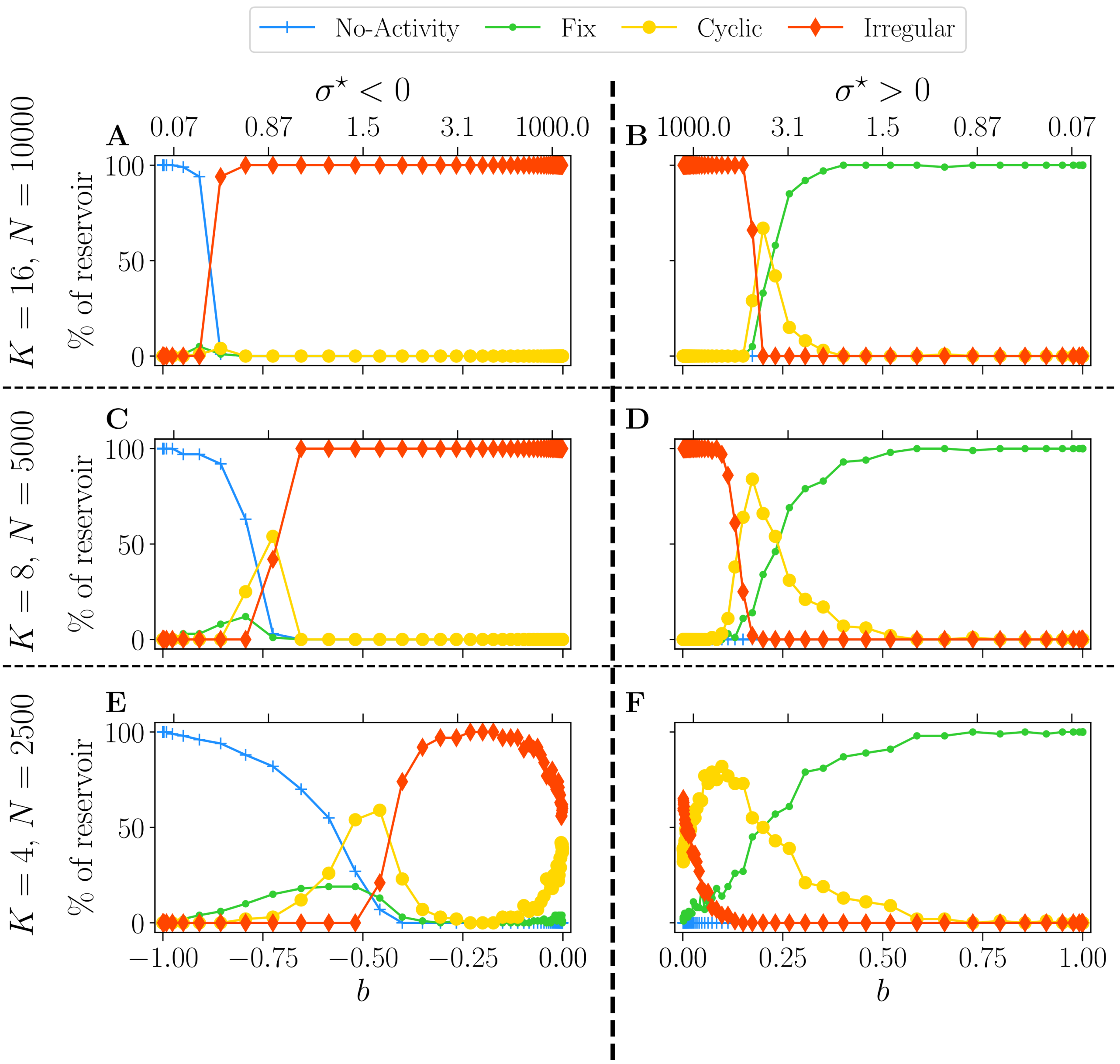

Figure 11. Attractor statistics of free-evolving RBN reservoirs, controlled by K and N with a constant ratio (rows), versus the balance b (x-axis). The lower x-axis represents the corresponding $|\sigma^\star|$, for $b < 0$ (**A**, **C**, **E**), and $b > 0$ (**B**, **D**, **F**). Each steady activity signal is classified into one of the four categories of attractors: no-activity (+), fix (•), cyclic (●), irregular (♦). The statistics of attractors are computed over 100 reservoirs run once (y-axis). Results are shown for $K = 16$, $N = 10000$ (**A** and **B**), $K = 8$, $N = 5000$ (**C** and **D**), and $K = 4$, $N = 2500$ (**E** and **F**).

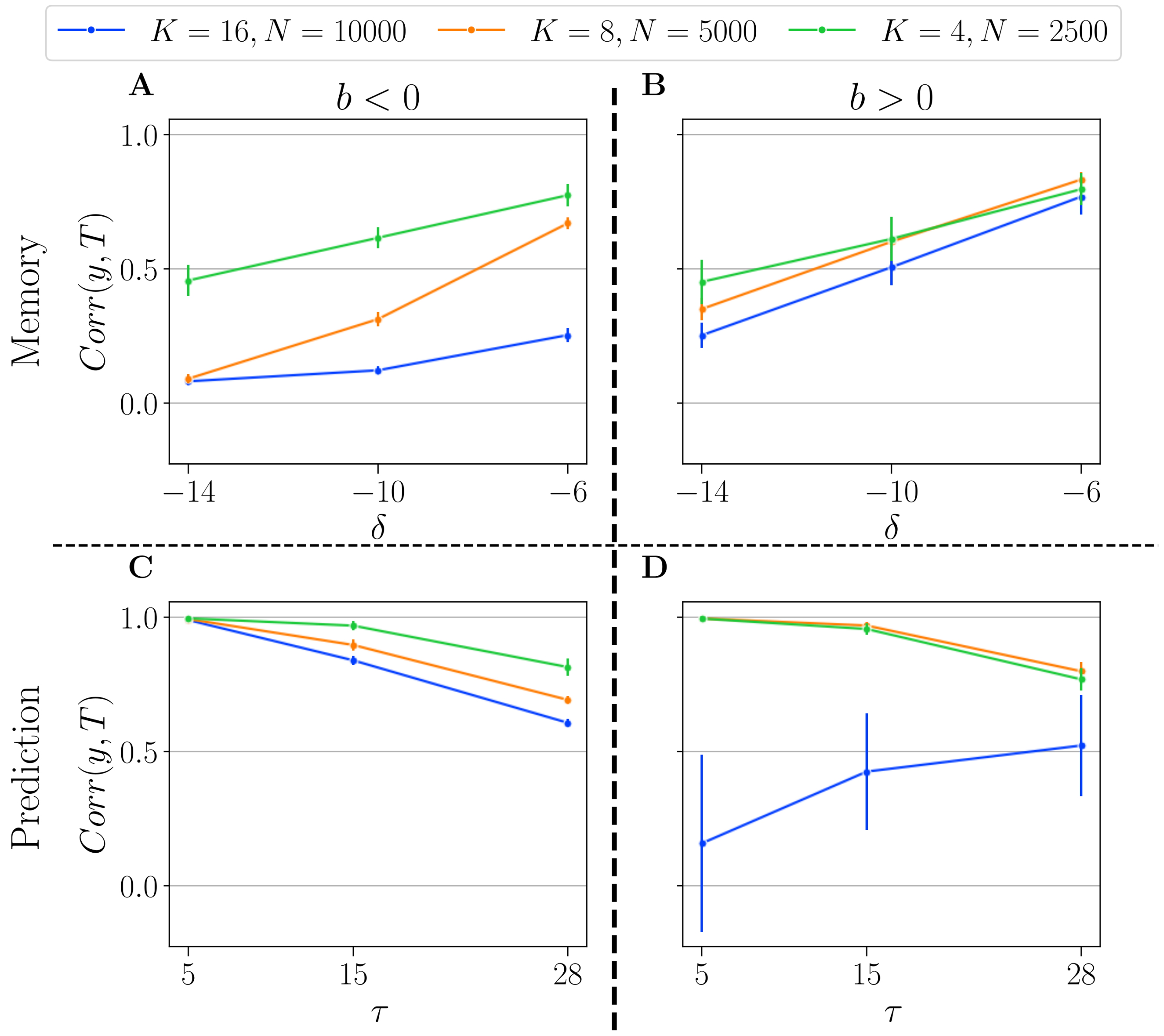

Figure 12. Summary of performance in the memory (**A** and **B**) and the prediction (**C** and **D**) tasks, for three couples of K and N resulting in the same density: $K = 4, N = 2500$; $K = 8, N = 5000$; and $K = 16, N = 10000$. Results are shown for both $b < 0$ (left panel), and $b > 0$ (right panel). For each value of (K, N), we selected the b_{opt} giving the highest average performance, in the most difficult task ($\delta = -14$ for memory, and $\tau = 28$ for prediction). We plot the performance (higher is better) of reservoirs $Corr(y, T)$ (y-axis), plotted as a function of their respective task parameters (x-axis): δ (memory) and τ (prediction). The solid line represents the average over 20 reservoirs (generated with the same b_{opt} and (K, N) values. The error bars represent one standard deviation.
